# Synergistic effects of *Bifidobacterium thermophilum* RBL67 and selected prebiotics on inhibition of *Salmonella* colonization in the swine proximal colon PolyFermS model

**DOI:** 10.1186/s13099-014-0044-y

**Published:** 2014-10-24

**Authors:** Sabine Amani Tanner, Christophe Chassard, Annina Zihler Berner, Christophe Lacroix

**Affiliations:** Laboratory of Food Biotechnology, Institute of Food, Nutrition and Health, Department of Health Science and Technology, ETH Zurich, Schmelzbergstrasse 7, 8092 Zurich, Switzerland

**Keywords:** *Bifidobacterium thermophilum* RBL67, Swine, Intestinal fermentation model, Prebiotics, Probiotics, *Salmonella enterica* subsp. *enterica* serovar Typhimurium N-15

## Abstract

**Background:**

Probiotics and prebiotics are promising strategies to counteract *Salmonella* prevalence in swine. In the present study, we investigated the effects of prebiotics (fructo- (FOS), galacto- (GOS) and mannan- (MOS) oligosaccharides) and the bacteriocinogenic *Bifidobacterium thermophilum* RBL67 (RBL67) on *Salmonella enterica* subsp. *enterica* serovar Typhimurium N-15 (N-15) colonization using the PolyFermS *in vitro* continuous fermentation model simulating the swine proximal colon.

**Material and methods:**

The PolyFermS model was designed with a first-stage reactor containing immobilized fecal pig microbiota. This reactor continuously inoculated five parallel second-stage reactors, a control and four treatment reactors, all operated with proximal colon conditions. FOS and GOS (5.2 g/day), and MOS (half dosage) and RBL67 (10^8^ copy numbers/mL applied daily) were tested on the ability of N-15 to colonize reactors, inoculated with the same microbiota. Reactor effluents were collected daily and analyzed for microbial composition (quantitative PCR and 454 pyrosequencing of 16S rRNA gene pool) and main metabolites (HPLC).

**Results:**

RBL67 and N-15 were shown to stably colonize the system. Colonization of N-15 was strongly inhibited by FOS and GOS, whereas addition of RBL67 alone or combined with MOS showed intermediate results. However, the effect of FOS and GOS was enhanced when prebiotics were combined with a daily addition of RBL67. FOS and GOS increased the total short chain fatty acid production, especially acetate and propionate. RBL67 combined with FOS additionally stimulated butyrate production.

**Conclusions:**

Our study demonstrates the suitability of the porcine PolyFermS *in vitro* model to study nutritional effects of pro- and prebiotics on gut microbiota composition and activity. It can further be used to monitor *Salmonella* colonization. The inhibition effects of FOS and GOS on N-15 colonization are partly due to an increased acetate production, while further antimicrobial mechanisms may contribute to an enhanced inhibition with prebiotic-RBL67 combinations. A future direction of this work could be to understand the anti-*Salmonella* effects of *Bifidobacterium thermophilum* RBL67 in the presence of prebiotics to unravel the mechanism of this probiotic:pathogen interaction.

**Electronic supplementary material:**

The online version of this article (doi:10.1186/s13099-014-0044-y) contains supplementary material, which is available to authorized users.

## Background

*Salmonella* are highly prevalent in swine where they affect about one third of all production holdings in the European Union [1]. *Salmonella* negatively impact pig health and the productivity of livestock. Transmission to humans occurs via the food-chain, leading to severe infections. Therefore, *Salmonella* control must be initiated at the farm level. Since antibiotics for growth promotion have been banned, alternative strategies to improve gut health are necessary to maintain productivity. Gut microbial composition and activity can be directly influenced via the diet [2]. This in turn impacts the colonization ability of enteric pathogens, such as *Salmonella,* through competitive exclusion mechanisms [3]. Probiotics and prebiotics, known for their potential to modulate gut microbial composition and activity, are amongst the promising alternative strategies [4].

Probiotics are defined as “live microorganisms which, when administered in adequate amounts, confer a health benefit on the host” [5]. Beneficial effects attributed to probiotics in pig feed include reduced incidence and severity of infections and decreased shedding of pathogens [6-8]. For example, weaned pigs treated with a five strain probiotic mixture (four *Lactobacillus* strains and one *Pediococcus* strain) showed significantly reduced (>2 log_10_ cfu/g feces) *Salmonella* numbers at 15 days post-infection [7]. Other authors report a lower incidence of diarrhea and fecal coliform numbers when feeding *Lactobacillus rhamnosus* GG [9], reduced carriage of *Escherichia coli* with *Bifidobacterium lactis* HN019 [10], or decreased *Salmonella* counts in feces and tissues after feeding pigs a combination of *Lactobacillus acidophilus* and *Lactobacillus reuteri* [8].

Prebiotics are non-digestible food-ingredients that are readily fermentable in the colon and stimulate potentially health-promoting bacteria, mainly bifidobacteria and/or lactobacilli, thereby beneficially shifting the microbial equilibrium of the host gut [11]. For example, Patterson et al. [12] reported stimulation of *Bifidobacterium* spp. and *Lactobacillus* spp. with a concomitant suppression of *Clostridium* spp. and members of *Enterobacteriaceae* spp. upon feeding of inulin to pigs. Prebiotics can stimulate short chain fatty acid (SCFA) production, known to play a key role in intestinal host health. For example, butyrate, the main energy source for colonocytes, has anti-inflammatory and anti-carcinogenic properties (reviewed by Russell et al. [13]) and down-regulates the expression of genes associated with *Salmonella* invasion [14]. However, conflicting results have been reported for the effects of prebiotic feeding in pigs. Tzortzis et al. [15] reported higher acetate concentrations and increased bifidobacteria numbers after feeding GOS to pigs, while Mikkelsen and Jensen [16] showed increased butyrate production after feeding FOS to piglets. In contrast, no effect was observed with FOS on bifidobacterial populations [17] and on fecal SCFA concentrations [18]. Prebiotics are increasingly combined with probiotics (synbiotics) to enhance probiotic survival and growth. Synbiotic formulations tested in pigs decreased the level of *Enterobacteriaceae* in pig fecal samples [19], and reduced adherence of *Escherichia coli* O8:K88 to the jejunal and colonic mucosa [20]. However, synbiotic formulations have been much less studied for pathogen inhibition. Yet, they have a promising potential considering the competitive advantage of the probiotic through simultaneous application of a prebiotic with high specificity [21,22].

The species *B. thermophilum* belongs to the commensals of the pig gut microbiota [23]. *Bifidobacterium thermophilum* RBL67 (RBL67) previously isolated from baby feces was shown to produce a bacteriocin-like substance (BLIS) with *in vitro* activity against *Listeria* and *Salmonella* [24-26]*.* Furthermore, we recently showed that RBL67 has antagonistic effects on *Salmonella* infection in an *in vitro* continuous intestinal fermentation model simulating the child proximal colon [27]. This strain was reported to adhere to human intestinal cell lines [28] and to exert protective effects on epithelial HT29-MTX cell culture integrity upon *Salmonella* challenge in combined cellular and colonic fermentation models [29]. Inulin supplemented in a three-stage continuous intestinal fermentation model of the child induced an increase of *B. thermophilum* numbers in the proximal, transverse and distal colon sections while SCFA production was shifted towards higher butyrate concentrations [30]. However, inulin in the proximal colon environment of the model was also shown to promote *Salmonella* growth [30], and to increase the efficiency of HT29-MTX cell invasion [29]. Finally, RBL67 has technological features of interest for application, such as being moderately oxygen-tolerant, growing at high cell density, low pH and high temperatures of up to 47°C [31].

Studying the complex interplay of pro- and prebiotics with the gut microbiota and pathogens is hindered by the inaccessibility of the gastrointestinal tract. Studies are further challenged by ethical limits to conduct *in vivo* animal infection trials. In this context, *in vitro* models represent a cost-effective and ethically less constraint strategy [32]. We recently reported and validated a novel two-stage *in vitro* continuous fermentation model (PolyFermS) inoculated with immobilized fecal microbiota simulating the swine proximal colon. This model allows the parallel operation of five self-contained independent fermentations to simultaneously test different nutritional factors with the same microbiota [33]. In this study, we used this PolyFermS model of the swine proximal colon to investigate the effects of *B. thermophilum* RBL67 and prebiotics (FOS, GOS and MOS) on the gut microbiota composition and activity and on the colonization of the enteric pathogen *Salmonella enterica* subsp. *enterica* serovar Typhimurium N-15 (N-15).

## Results

### *Colonization potential* of RBL67 and N-15

**Figure 1:**
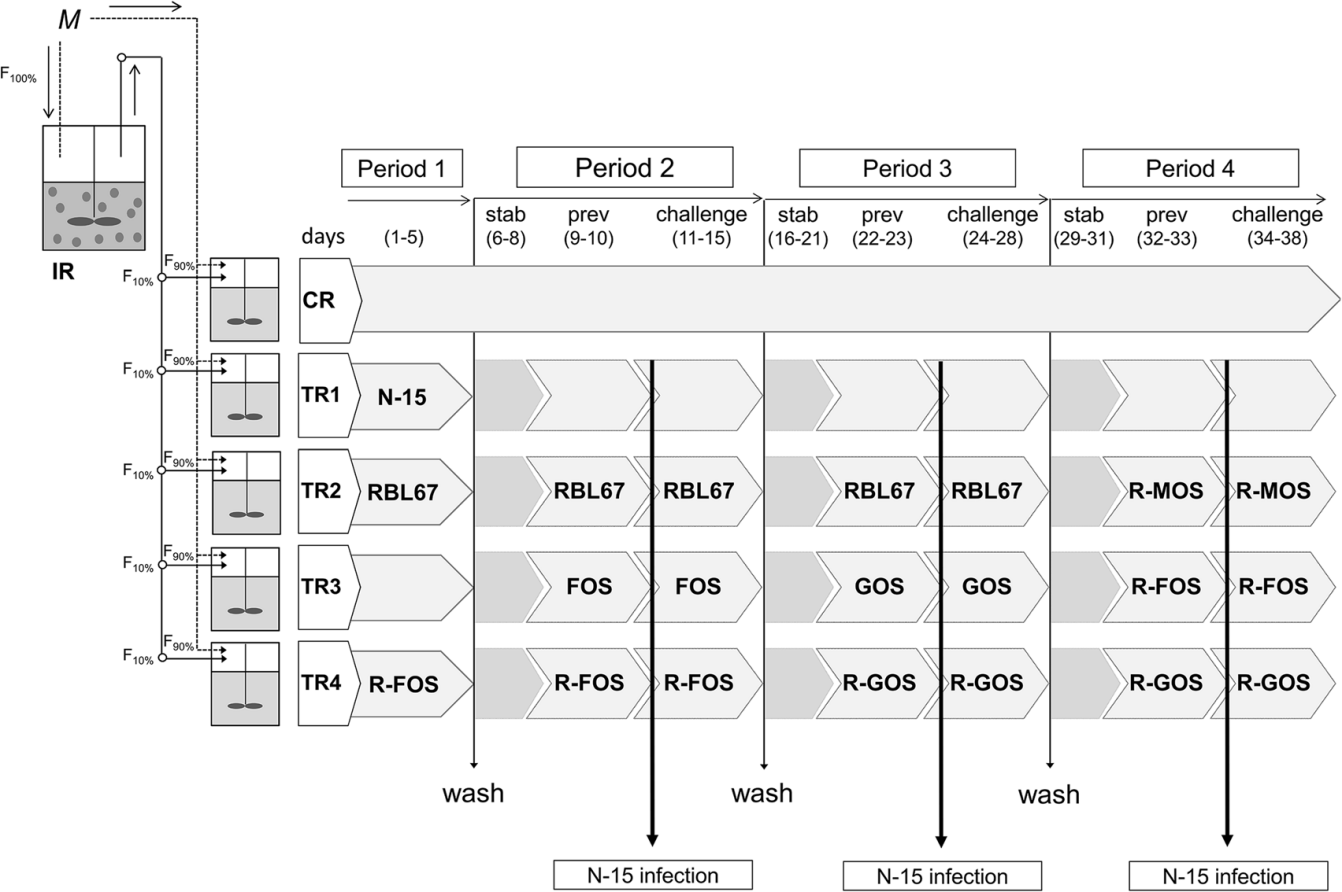
**Experimental set-up of the continuous fermentation experiment.** IR: inoculum reactor; CR: control reactor; TR: test reactors 1–4; F: flow rate; *M:* fresh medium inflow; stab: stabilization; prev: prevention; challenge: challenge with *Salmonella* N-15; N-15: *S.* Typhimurium N-15; RBL67: *B. thermophilum* RBL67; R-FOS/GOS/MOS: *B. thermophilum* RBL67 + respective prebiotic.

**Figure 2:**
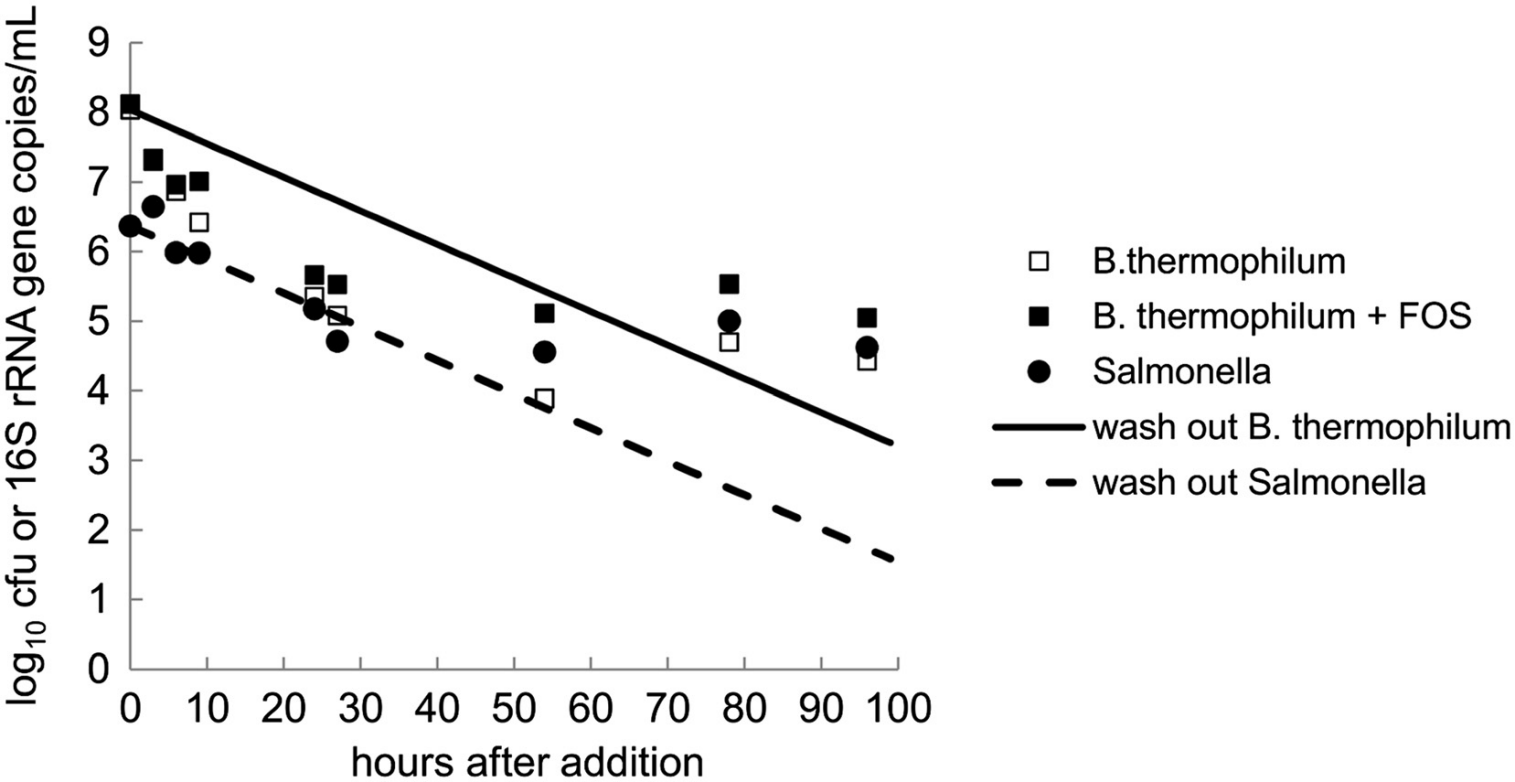
***Salmonella*** **and** ***B. thermophilum*** **in reactor effluents compared to theoretical washout curves during colonization tests.** RBL67 was added once to TR2 and TR4 to reach 10^8^ CN/mL, while TR4 was additionally supplied with 5.2 g of FOS/day. N-15 was added once to TR1 to reach 10^6^ cfu/mL. *Salmonella* viable cell counts in reactor effluents was measured by plating on CHROMAgar™. *B. thermophilum* numbers were estimated by qPCR. Measured concentrations were compared to a theoretical washout curve.

To evaluate the colonization ability of RBL67 and N-15 in an *in vitro* model of the swine proximal colon, we inoculated TRs once with RBL67 with and without FOS or with N-15 during period 1 (Figure 1). RBL67 and N-15 concentrations were estimated 96 h after addition and data were compared to the theoretical washout curve (Figure 2).

The N-15 cell counts initially declined at a rate close to the theoretical washout curve and stabilized after 27 h at 4.7 ± 0.2 log_10_ cfu/mL until 96 h. RBL67 gene copy numbers (CN) (8.1 log_10_ CN/mL) declined faster than the theoretical washout curve during the first 54 h and reached a stable value of 4.6 ± 0.2 log_10_ CN/mL between 78 and 96 h. A similar pattern was observed for the treatment of RBL67 combined with FOS, with CN decreasing until 27 h, followed by stability (5.3 ± 0.3 log_10_ CN/mL, 27–96 h).

### Effect of prebiotics and RBL67 on N-15 colonization

**Figure 3:**
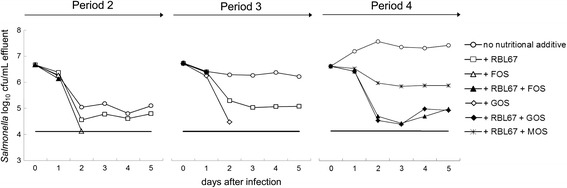
***Salmonella*** **cell counts determined in test reactors during treatment periods 2–4.** Treatment periods 2–4 were used to test the effect of prebiotics (FOS, GOS) or RBL67 or combinations of RBL67 with FOS, GOS or MOS on *Salmonella* N-15 colonization. RBL67 and/or prebiotics were added to TRs at 10^8^ CN/mL and 5.2 g/day, respectively during a 2 days prevention period and for 5 days after N-15 challenge. N-15 was inoculated once at 10^6^ cfu/mL and was monitored by plate counts on CHROMAgar™ *Salmonella*. (─) *Salmonella* detection limit of 4.1 cfu/mL. Cell counts at day 0 correspond to the inoculum added to the reactors.

Pretreatments with RBL67 and prebiotics were tested during periods 2–4 on N-15. After N-15 infection in period 2, N-15 cell counts declined 1.6 log_10_ cfu/mL during the first 2 days and stabilized at 5.0 ± 0.2 log_10_ cfu/mL effluent (days 2–5) (Figure 3). Unexpectedly, N-15 cell counts in the following periods showed either a limited initial decline phase after the first day of challenge followed by stability (6.3 ± 0.1 log_10_ cfu/mL, period 3, days 1–5), or a steady increase until day 2 to reach 7.4 ± 0.1 log_10_ cfu/mL (period 4, days 2–5). The treatments with FOS and GOS during periods 2 and 3 induced a strong inhibition of N-15 colonization, with N-15 cell numbers decreasing below the detection limit (4.1 log_10_ cfu/mL effluent) 3 days post-infection. When FOS or GOS were combined with RBL67 (R-FOS and R-GOS) during periods 2–4, N-15 counts decreased even more rapidly compared to treatments with the prebiotics alone, reaching non-detectable levels after two days post-infection (periods 2 and 3) or reducing initial N-15 counts by approximately 2 log_10_ cfu/mL (period 4). Intermediate effects were recorded for RBL67 alone (periods 2 and 3) and in combination with MOS (R-MOS, period 4), with a reduction of N-15 counts 2 days post-infection by approximately 1.8 ± 0.3 and 0.7 log_10_ cfu/mL, respectively.

### Effect of prebiotics, RBL67 and N-15 infection on gut microbiota composition

Changes in the microbial community composition were monitored by qPCR and by 454 pyrosequencing. We compared mean copy numbers (days 2–4) of bacterial populations during pseudo-steady states of N-15 challenge periods of control and treatment reactors.

*Bacteroides-Prevotella* and *Clostridium* Cluster IV were the most prominent groups, followed by *Enterobacteriaceae, Lactobacillus/Leuconostoc/Pediococcus* and *Bifidobacterium* (Table 1). Total 16S rRNA, *Clostridium* Cluster IV and *Bacteroides*-*Prevotella* gene copy numbers remained stable independent of the tested conditions. Furthermore, the other bacterial groups, except for *Bifidobacterium*, did not show large changes (difference to CR <0.5 log_10_ CN/mL) upon treatment application. *Bifidobacterium* numbers increased by more than 1 log_10_ CN/mL during treatments with RBL67 alone and RBL67 combined with prebiotics (R-FOS, R-GOS, R-MOS). *B. thermophilum* was detected during daily treatments with RBL67, at concentrations ranging from 7.6 and 8.1 log_10_ CN/mL, but not in the other treatments and in CR (Table 1). The highest numbers of *B. thermophilum* were measured for RBL67 and FOS applied in combination (R-FOS). N-15 inoculation in absence of dietary treatments showed no effect on *Enterobacteriaceae* numbers, but was associated with a slight but significant increase of the group *Lactobacillus/Leuconostoc/Pediococcus* (0.6 log_10_ CN/mL, period 2) and of *Bifidobacterium* (0.6 log_10_ CN/mL, period 4) compared to CR.

**Table 1:** **16S rRNA gene copy numbers of bacterial groups by qPCR in reactors during periods 2-4**

	**Bacterial group, log**_**10**_ **copies/mL effluent**						
**Treatment**	***Bifidobacterium thermophilum***	***Bifidobacterium*** **spp.**	***Bacteroides-Prevotella***	***Enterobacteriaceae***	***Lactobacillus/Leuconostoc/Pediococcus*** **spp.**	***Clostridium*** **Cluster IV**	**total 16S rRNA genes**
PERIOD 2							
**Control**	n.d.	6.4 ± 0.2	10.3 ± 0.1	9.6 ± 0.1	7.6 ± 0.1	10.0 ± 0.1	10.5 ± 0.1
**N-15**	n.d.	6.5 ± 0.2	10.3 ± 0.03	9.6 ± 0.1	8.2 ± 0.3^*^	10.1 ± 0.1	10.5 ± 0.2
**RBL67**	7.6 ± 0.1	8.3 ± 0.2^*^	10.2 ± 0.1	10.0 ± 0.1^*^	7.6 ± 0.2	10.0 ± 0.1	10.6 ± 0.2
**FOS**	n.d.	6.0 ± 0.2^*^	10.4 ± 0.04	9.9 ± 0.2^*^	7.4 ± 0.1	10.0 ± 0.03	10.7 ± 0.1
**R-FOS**	8.1 ± 0.1	8.7 ± 0.2^*^	10.2 ± 0.2	9.9 ± 0.2	7.4 ± 0.2^*^	10.0 ± 0.1	10.7 ± 0.1
PERIOD 3							
**Control**	n.d.	6.7 ± 0.1	10.0 ± 0.03	10.0 ± 0.1	8.2 ± 0.3	10.0 ± 0.02	10.6 ± 0.1
**N-15**	n.d.	6.9 ± 0.2	10.1 ± 0.2	9.8 ± 0.1^*^	8.2 ± 0.2	10.1 ± 0.2	10.6 ± 0.2
**RBL67**	7.7 ± 0.1	7.7 ± 0.1^*^	10.1 ± 0.2	10.2 ± 0.1^*^	8.6 ± 0.2	10.1 ± 0.1	10.6 ± 0.2
**GOS**	n.d.	6.6 ± 0.04	9.9 ± 0.2	9.7 ± 0.2^*^	8.1 ± 0.2	10.0 ± 0.5	10.5 ± 0.1^*^
**R-GOS**	7.7 ± 0.1	8.0 ± 0.1^*^	10.0 ± 0.2	9.9 ± 0.2	8.3 ± 0.3	10.1 ± 0.3	10.5 ± 0.2
PERIOD 4							
**Control**	n.d.	6.4 ± 0.1	10.0 ± 0.2	10.0 ± 0.2	8.0 ± 0.2	9.5 ± 0.02	10.6 ± 0.3
**N-15**	n.d.	7.0 ± 0.1^*^	9.9 ± 0.1	9.8 ± 0.1	8.1 ± 0.3	9.8 ± 0.1	10.5 ± 0.1
**R-MOS**	7.6 ± 0.1	8.1 ± 0.1^*^	9.8 ± 0.3	9.8 ± 0.2	8.2 ± 0.4	9.5 ± 0.3	10.5 ± 0.4
**R-FOS**	8.0 ± 0.1	8.4 ± 0.1^*^	9.8 ± 0.1	9.9 ± 0.2	8.4 ± 0.2^*^	9.7 ± 0.1	10.5 ± 0.1
**R-GOS**	7.8 ± 0.1	8.3 ± 0.03^*^	9.9 ± 0.1	10.0 ± 0.2	8.3 ± 0.2	9.8 ± 0.2	10.6 ± 0.3

^*^Bacterial populations significantly different (P < 0.05) from CR within a period; Values are given as means ± SD (log_10_ copy numbers/mL reactor effluent) calculated from three consecutive days (days 2–4) during N-15 challenge periods.

N-15: *Salmonella* N-15 without pro- or prebiotic; RBL67: *B. thermophilum* RBL67 alone; FOS: FOS alone; R-FOS: *B. thermophilum* RBL67 + FOS; GOS: GOS alone; R-GOS: *B. thermophilum* RBL67 + GOS; R-MOS: *B. thermophilum* RBL67 + MOS.

**Figure 4:**
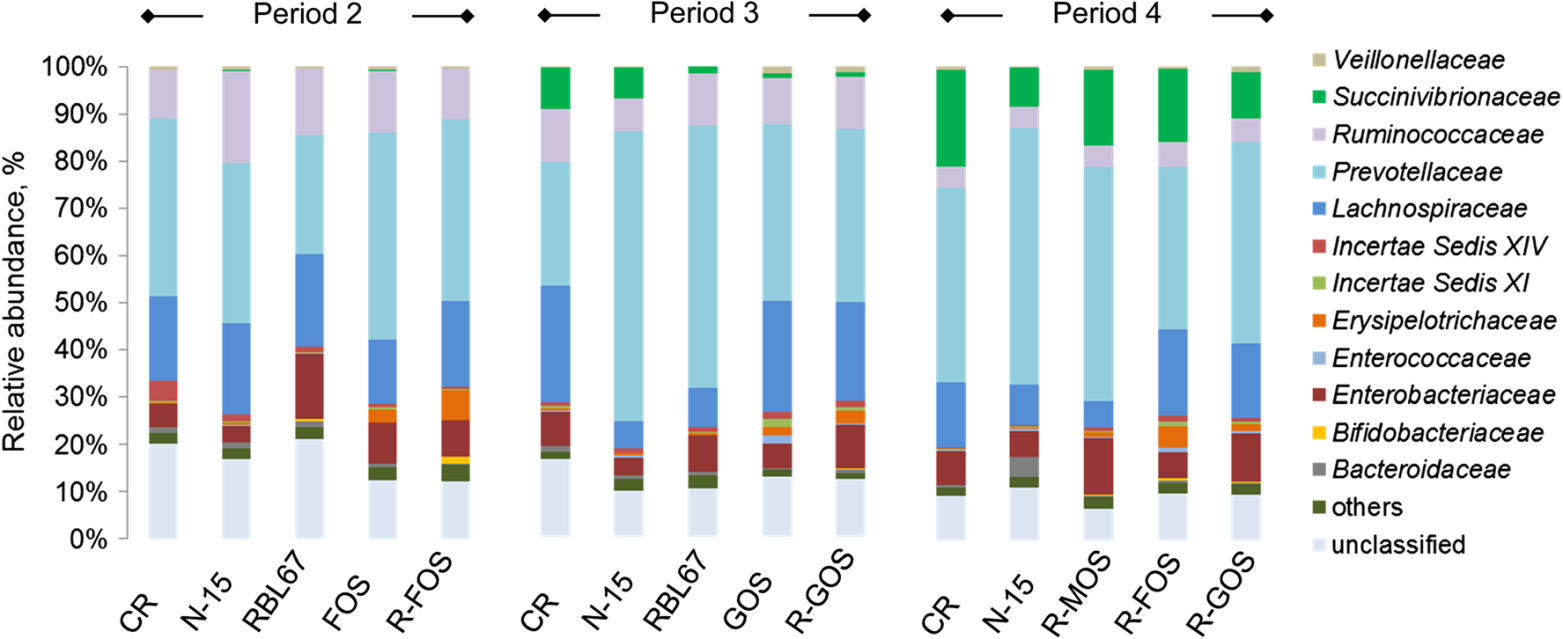
**Microbial composition in reactors during treatment periods 2–4 measured by 454 pyrosequencing on family level.** The microbiota profile in reactor effluents during treatment periods was analyzed by 454 pyrosequencing of the entire 16S rRNA gene pool in the V5-V6 region. Reactor effluents were pooled in a ratio 1:1 from two consecutive days of the N-15 challenge period (days 3 and 4) for genomic DNA extraction and subsequent sequencing on a 454 Life Sciences Genome Sequencer GS FLX instrument. Quality-filtered sequencing reads were assigned using the Ribosomal Database Project (RDP) Bayesian classifier (v2.1) and applying a confidence threshold of 80%. CR: control reactor; values <1% are summarized in the group others.

Using 454 pyrosequencing of the entire 16S rRNA gene pool, a mean value of 6259 ± 3730 quality-filtered reads per sample was obtained with an average read length of 256 ± 1 bp. All samples revealed the predominance of the 3 phyla, Firmicutes, Bacteroidetes and Proteobacteria (Additional file 1: Figure S1). Additionally, Actinobacteria were detected at low levels (<1%; except for R-FOS in period 2 with 1.9%). Firmicutes and Bacteroidetes accounted for more than 80% of assigned reads in all samples for periods 2 and 3. However, during period 4, Proteobacteria increased to up to 30% while Firmicutes and Bacteroidetes decreased to approximately 70% of all reads. The phylum Proteobacteria displayed a steady increase in all reactors during the fermentation, including in CR where no treatment was applied. In general, pro- and prebiotic treatments and N-15 infection did not markedly impact microbiota composition. At the phylum level, Bacteroidetes increased and Firmicutes decreased in the N-15 (alone) and RBL67 treatments compared to CR (period 3). On the family level a consistent increase of *Erysipelotrichaceae* was observed with prebiotics, alone (FOS, GOS) or in combination with RBL67 (R-FOS, R-FOS, R-MOS), compared to CR, with highest effect for R-FOS (6.4% compared to 0.3% in CR, period 2 and 4.7% compared to 0.1% in CR, period 4) (Figure 4). Changes observed at the genus level (Additional file 2: Figure S2) were consistent with observations at the family level. The genus *Sharpea*, a member of the family *Erysipelotrichaceae,* was highly abundant in the TRs after FOS or GOS treatments and the combined treatments of RBL67 with prebiotics (R-FOS, R-GOS, R-MOS) compared to CR. The genus increased to 6% (period 2) and 5% (period 4) of total reads after the R-FOS treatment with values < 0.1% in CR.

### Effect of prebiotics, RBL67 and N-15 infection on gut microbiota metabolism

Metabolite concentrations were measured by HPLC and mean values of three consecutive days (days 2–4), corresponding to pseudo-steady states of the N-15 challenge periods, were compared to corresponding data from the CR (Table 2).

**Table 2:** **Concentration (mM) and molar ratios (%) of metabolites measured by HPLC during periods 1-4**

	**Acetate**	**Propionate**	**Butyrate**	**total SCFA**	**ratio (acetate:propionate:butyrate)**
PERIOD 1					
**Control**	94.6 ± 2.3	45.8 ± 1.3	22.5 ± 0.4	162.9 ± 1.8	58:28:14
**N-15**	104.2 ± 1.0*	43.6 ± 1.5	22.9 ± 1.1	170.6 ± 3.0*	61:26:13
**RBL67**	96.8 ± 6.1	43.0 ± 0.3*	25.7 ± 1.0*	165.5 ± 5.4	58:26:16
**R-FOS**	146.8 ± 5.9*	52.1 ± 3.3*	26.4 ± 0.9*	225.3 ± 1.8*	65:23:12
PERIOD 2					
**Control**	92.4 ± 3.7	48.0 ± 2.0	20.7 ± 0.8	161.0 ± 5.1	57:30:13
**N-15**	94.0 ± 6.9	45.2 ± 1.1	21.9 ± 1.8	161.1 ± 8.2	58:28:14
**RBL67**	102.3 ± 2.6*	44.3 ± 1.3*	24.3 ± 1.3*	170.9 ± 5.0*	60:26:14
**FOS**	126.4 ± 5.2*	58.4 ± 1.1*	21.0 ± 2.4	205.8 ± 6.0*	61:28:10
**R-FOS**	112.2 ± 1.0*	60.6 ± 0.9*	29.9 ± 1.1*	202.7 ± 1.5*	55:30:15
PERIOD 3					
**CR**	89.5 ± 1.8	46.9 ± 0.1	27.0 ± 1.8	163.4 ± 3.6	55:29:16
**SAL**	86.1 ± 1.4*	50.0 ± 2.8	22.9 ± 5.2*	159.0 ± 5.2	54:31:14
**RBL67**	101.3 ± 3.1*	47.3 ± 2.0	22.7 ± 5.9*	171.3 ± 5.9	59:28:13
**GOS**	122.7 ± 4.8*	61.1 ± 5.5*	21.6 ± 5.0*	205.4 ± 5.0*	60:30:10
**R-GOS**	117.6 ± 2.0*	60.4 ± 2.7*	27.1 ± 5.9	205.1 ± 6.0*	57:29:13
PERIOD 4					
**CR**	78.7 ± 0.8	51.4 ± 1.0	23.2 ± 0.4	153.3 ± 1.4	51:34:15
**SAL**	90.1 ± 1.0*	50.9 ± 0.9	22.8 ± 1.7	163.8 ± 1.7*	55:31:14
**R-MOS**	82.7 ± 3.2*	64.1 ± 1.5*	22.8 ± 2.9	169.3 ± 1.0*	49:38:13
**R-FOS**	114.6 ± 7.6*	65.0 ± 1.3*	24.7 ± 0.6*	204.3 ± 6.0*	56:32:12
**R-GOS**	118.5 ± 1.1*	68.2 ± 3.0*	20.1 ± 0.3*	206.8 ± 2.9*	57:33:10

*Means significantly different (P < 0.05) from CR within a metabolite and period using the Kruskal-Wallis pairwise comparison.

Reported are mean values (days 2–4 of N-15 challenge periods) ± SD and ratios are given as percentage of total SCFA (acetate, propionate, butyrate).

N-15: *S.* Typhimurium N-15 without pro- or prebiotic; RBL67: *B. thermophilum* RBL67 alone; FOS: FOS alone; R-FOS: *B. thermophilum* RBL67 + FOS; GOS: GOS alone; R-GOS: *B. thermophilum* RBL67 + GOS; R-MOS: *B. thermophilum* RBL67 + MOS.

In the CR, the total short chain fatty acid (SCFA) concentration was stable from periods 1 to 3 (162 ± 1 mM), but a slight decrease to 153 ± 1 mM was observed during period 4, corresponding to a switch of the metabolite molar ratio (acetate:propionate:butyrate) from 57:29:14 (period 1–3) to 51:34:15 (period 4). The total SCFA concentration in the TRs was increased by 29 ± 4% compared to the CR for treatments with FOS and GOS alone and combined with RBL67. Acetate (+38 ± 10%) and propionate (+28 ± 4%) levels were most increased with FOS, GOS, R-FOS and R-GOS, whereas R-FOS also induced a significant increase of butyrate (18%, 45% and 7% for period 1, 2 and 4, respectively) compared to the CR. The total SCFA concentration was also significantly increased in TRs treated with RBL67 alone (+5 ± 1%, periods 2–3) or with R-MOS (+11%), although to a lesser extent than for the other prebiotics. Treatment with RBL67 increased acetate (+12 ± 2%, periods 1–2) and butyrate concentrations (+16 ± 2%, periods 1–2), while R-MOS mainly stimulated propionate production (+25%). Infection with N-15 (alone) had little effect on metabolite productions, except for an increase in acetate concentration (+11 ± 2%, periods 1 and 4). Branched chain fatty acids (BCFA) were measured at low amounts (<7 mM) in all reactors. Formate and lactate were not detected throughout the fermentation (data not shown).

## Discussion

We recently described and validated a novel *in vitro* continuous fermentation model (PolyFermS) simulating conditions of the swine proximal colon. The model consists of parallel reactors inoculated with the same microbiota [33]. In this study, we report the first time application of this swine PolyFermS model to investigate the effects of a probiotic strain, *B. thermophilum* RBL67, prebiotics (FOS, GOS, MOS) and combinations thereof, on *S.* Typhimurium N-15 colonization in the presence of a diverse gut microbiota.

In a first test, RBL67 and N-15 were shown to colonize the system after one single inoculation. They reached stable and similar numbers after 1 to 2 days. Our *in vitro* model data suggest competitive and adaptive traits of RBL67 and N-15 in co-culture with the modeled porcine microbiota. These results are in agreement with previous studies done with one- and three-stage chemostat models of the child colon [27,34]. The increasing capacity of N-15 to colonize the model observed from periods 2 to 4, underlines the robustness and/or adaptation of *Salmonella* in simulated colonic conditions of the swine colon. This suggests that the PolyFermS model is suitable to mimic a *Salmonella* carrier state of pigs with continuous shedding of *Salmonella* [35]. Moreover, an incomplete removal of N-15 during washing periods of reactors may partly explain the enhanced competition of N-15 over time, because viable cells of *Salmonella* were detected in the effluents by plating after careful washing with 10% chlorine for 1 h and prior to N-15 challenge in periods 3 and 4 (data not shown). This persistence of *Salmonella* could be due to the formation of biofilms in the reactor, which is known to increase sterilization resistance [36]. This effect may be avoided in the future by replacing the test reactors with sterile units before each new treatment period. We also reported an increase of the family *Succinivibrionaceae* during the course of the fermentation for the first-stage immobilized cell and all second-stage reactors for the same fermentation test [33]. *Salmonella* and *Succinivibrionaceae* belong to the γ-subclass of the phylum Proteobacteria [37]. Increased numbers of *Succinivibrionaceae* correlated with the increased capacity of N-15 to grow in the system, suggesting that this group potentially supported N-15 persistence and growth in periods 3 and 4 after washing. Such co-occurrence of related bacteria has been previously reported for *Salmonella* invasion in a mouse infection model in the presence of high titers of *E. coli* [38].

Colonization of N-15 in the porcine PolyFermS was strongly inhibited by the addition of FOS or GOS. This correlated with an increase of SCFA production, especially acetate and propionate. A 5 mM undissociated acetic acid solution was reported to inhibit *Salmonella* growth [39-41]. In our study, concentrations of undissociated acetic acids were calculated to be >6 mM (pH = 6.0) for treatments with FOS and GOS, compared to levels ≤5 mM in the reactor spiked with N-15 alone. RBL67 combined with FOS or GOS showed an enhanced inhibition of N-15 compared to single treatments with pro- or prebiotics. We chose strain RBL67, because it produces BLIS (thermophilicin B67), which exhibits an antagonistic effect against *Salmonella* and *Listeria* [24-26]. The production of acetate was decreased for R-FOS and R-GOS compared to prebiotics alone (Table 2). This suggests that BLIS contributed to N-15 inhibition in combination with organic acids produced by FOS and GOS. The lower dosage of the prebiotic in R-MOS compared to the other combinations and the stimulation of propionate rather than acetate production, may explain the less pronounced effect on N-15 colonization. However, MOS has previously been shown to block enteropathogen adhesion to the mannose-rich surface glycoproteins of epithelial villi via binding of its α-D-Mannan to Type 1 fimbriae of enteropathogens and thus may reduce the risk of infection by this mechanism [42].

The antagonistic effect of RBL67 was less pronounced in this study compared to a previous report [27]. A strong inhibition of *Salmonella* and a rapid metabolic rebalancing of the gut microbiota after antibiotic treatments were observed when RBL67 was added before or after infection in an *in vitro* intestinal fermentation model inoculated with child microbiota [27]. In contrast, Zihler et al. [30] did not detect an anti-*Salmonella* effect of RBL67. This may be explained by different host microbiota, model set-up and probiotic:pathogen ratios used for all these studies, i.e. 16:1 (this study), 3050:1 [27] and 2:1 [30].

FOS has been reported to stimulate butyrate production in some studies with piglets [16,43]. In our study, we observed an increased butyrate production with the combination of FOS and RBL67. Because bifidobacteria do not produce butyrate [44], we presume that FOS was first degraded e.g. by RBL67, followed by cross-feeding reactions with butyrate-producing bacteria (e.g. *Roseburia* spp. or *Megasphaera*; [45]). Interestingly, while butyrate has been linked to a series of health-related properties (reviewed by Russell et al. [13]), it was also shown to repress invasion gene expression of *Salmonella* [14].

The microbiota composition from CR to TR effluents only changed marginally after RBL67 and prebiotic treatments. In particular, we did not observe a growth stimulation of bifidobacteria or lactobacilli in the FOS and GOS treatments, as it was previously shown *in vitro* with human gut microbiota treated with FOS and inulin [30,46] or pig microbiota treated with GOS [15,47]. Divergent results have been reported concerning the effect of FOS and GOS *in vivo.* Patterson *et al.* [12] reported increased numbers of bifidobacteria and lactobacilli in young pigs fed with inulin. In contrast, Mountzouris et al. [17] and Mikkelsen and Jensen [16] did not observe a significant stimulation of bifidobacteria and lactobacilli in pigs fed with FOS and transgalactooligosaccharides. These discrepancies may be explained by different prebiotic structures, dosage and methodology [4,48], complicating a direct comparison between the studies. Furthermore, other bacteria of the gut microbiota, including *Salmonella* and members of *Roseburia* and *Bacteroides*, can efficiently utilize FOS and GOS as growth substrates [49-51] and can directly compete for these nutrients with bifidobacteria and lactobacilli.

Using 454 pyrosequencing, we detected a consistent increase in the relative abundance of the genus *Sharpea* upon addition of prebiotics. This suggests that *Sharpea* spp*.* play a role for prebiotic degradation. They belong to the family *Erysipelotrichaceae* within the *Clostridium* Cluster XVII. Members of this genus are heterofermentative and produce lactic acid and CO_2_ from glucose. They were first isolated from horse feces and are closely related to *Eggerthia catenaformis* [52,53]. Higher net substrate availability upon prebiotic addition may be responsible for a higher abundance of *Sharpea* spp*. Erysipelotrichaceae* were also more abundant in pigs with increased feed consumption [54,55], and accounted for a sevenfold higher proportion in mice fed a high energy diet [56]. Yet, the exact role of the genus *Sharpea* remains unclear and further insights into prebiotic degradation or its involvement in possible cross-feeding reactions should be elucidated in future research.

## Conclusion

Our data highlight the suitability of the novel porcine PolyFermS model to discover ecophysiological changes resulting from different nutritional treatments on *S.* Typhimurium N-15 colonization. We showed that FOS and GOS distinctively inhibit N-15 colonization in this model, while the effect was enhanced in presence of *B. thermophilum* RBL67. This was likely due to a combined effect of SCFA and antimicrobial compound production and competition. We showed that RBL67 stimulates butyrate production in the presence of FOS, beneficially impacting swine gut health. Future research should thus focus on elucidating the antagonistic mechanisms of RBL67 towards N-15 in the presence of prebiotics such as FOS and GOS.

## Methods

### Bacterial strains

*B. thermophilum* RBL67 (LMG S-23614, Laboratory of Food Biotechnology, ETH Zurich) was isolated from human baby feces [26]. *S.* Typhimurium N-15 was obtained from a clinical case and was supplied by the National Center for Enteropathogenic Bacteria and *Listeria* (NENT; University of Zurich, Zurich, Switzerland). RBL67 and N-15 were cultured from a glycerol stock (33%, −80°C) in serum flasks containing the fermentation medium used to simulate swine chyme [33], at 37°C for 15 h. The headspace of the serum flasks was flushed with an N_2_:CO_2_ (3:1) gas mixture before autoclaving to generate anaerobic conditions. Viable cell counts of *Salmonella* were determined by plating serial 10-fold dilutions in duplicate on CHROMAgar™ *Salmonella* (Becton Dickinson AG, Allschwil, Switzerland).

### Prebiotics

Fibrulose F97 (FOS) (Cosucra Groupe Warcoing S. A., Warcoing, Belgium) contains oligofructose (≥97% [wt/wt]) and minor amounts of free fructose, glucose and sucrose (≤5% [wt/wt]), and has a polymerization degree of 94% ≤20. Vivinal GOS 90 (GOS), composed of 96.5% GOS, 2% lactose, 0.7% glucose and 0.8% galactose, was supplied by Friesland Campina Domo (Amersfoort, Netherlands). Bio-Mos (MOS) was obtained from Alltech (Sarney, Ireland).

### Fermentation set-up

The experimental set-up of the continuous *in vitro* fermentation model was presented in detail by Tanner et al. [33]. Briefly, the fermentation model consisted of a two-stage reactor set-up, with six reactors operated under conditions of the swine proximal colon (38°C, pH 6.0, retention time 9 h, anaerobiosis by CO_2_ headspace flushing) (Figure 1). The inoculum reactor (IR) containing 30% (v/v) polysaccharide gel beads immobilizing swine fecal microbiota was used to continuously inoculate five subsequent reactors (one control (CR) and four test reactors (TR1-4)) with 10% effluent. CR and TR1-4 were additionally fed with 90% fresh nutritive medium, designed to simulate swine chyme [33]. While IR and CR were operated under constant conditions during the entire fermentation period, the test reactors (TR1-4) were used to test N-15 and RBL67 colonization (period 1) and effects of RBL67 and/or prebiotics on N-15 colonization (periods 2–4) (Figure 1)*.* Between each period, test reactors were disconnected from the IR, washed with 10% chlorine solution, reconnected and microbiota composition and activity was re-established for minimum 3 days before application of a new treatment [33].

### Period 1: RBL67- N-15 colonization

Colonization of *S.* Typhimurium N-15 and *B. thermophilum* RBL67 was tested during period 1 (Figure 1). N-15 was inoculated in TR1 once to reach a cell concentration of 10^6^ cfu/mL reactor. RBL67 was added once to TR2 and TR4 for a final gene copy number of 10^8^ CN/mL, while TR4 was additionally supplied with 5.2 g of FOS/day. Effluent samples were analyzed after 3, 6, 9, 24, 27, 54, 78 and 96 h for enumeration of *Salmonella* and *B. thermophilum* with plate counts and qPCR, respectively*.* Measured concentrations of N-15 and RBL67 were compared to a theoretical washout curve, calculated with the formula: c_t_ = c_0_* e^(−t/RT)^, where RT is the mean retention time (9 h), c_0_ and c_t_ are cell concentrations of bacteria at time point 0 and t, respectively.

### Periods 2–4: N-15 treatment periods

The effects of RBL67, FOS, GOS and combinations of RBL67 with FOS (R-FOS), GOS (R-GOS) and MOS (R-MOS) on N-15 colonization were tested during periods 2–4. For each period one reactor served as control (CR) and one reactor was infected with N-15 only (Figure 1). Treatment periods were divided into three phases: stabilization (stab) was carried out for 3 days (periods 2 and 4) or 5 days (period 3), prevention (prev) with pro- and/or prebiotics was done for 2 days, and challenge with N-15 was tested for 5 days, while addition of RBL67 and/or prebiotics was pursued. During prevention and challenge periods RBL67 and prebiotics were applied daily (Figure 1). All test reactors were infected once with N-15 on the first day of the challenge period.

RBL67 and N-15 inoculum was prepared from an overnight culture, which was centrifuged (6000 × g, 5 min) and resuspended in fresh fermentation medium. Reactors were inoculated with a syringe to obtain final concentrations of approximately 10^8^ CN/mL for RBL67 and 10^6^ cfu/mL for N-15 corresponding to a probiotic: pathogen ratio of approximately 100:1. FOS and GOS were supplied twice daily for a total of 5.2 g/day. This addition level was selected to correspond to approximately 3% (w/w) of the daily feed for pigs, considering a 2 kg/d feed intake and a scale factor of 0.09 for the ratio of the reactor volume (260 mL) to the pig proximal colon volume *in vivo* (approx. 2.9 L [57]). MOS was supplied only once per day and at 1.5% (w/w, 2.6 g/day), because higher amounts led to blocking of the flow. Reactor effluent samples were collected daily during the entire fermentation and analyzed for bacterial composition and activity.

### qPCR analyses

Predominant bacterial groups of the swine gut microbiota [58] in reactor effluents were enumerated by qPCR. Genomic DNA was extracted using the FastDNA Spin Kit for soil (MP Biomedicals, Illkirch, France) according to the manufacturer’s instructions. qPCR targets were: total bacteria (total 16S rRNA gene copies), *Bacteroides-Prevotella* group, *Enterobacteriaceae, Lactobacillus/Pediococcus/Leuconostoc* spp., *Clostridium* Cluster IV and *Bifidobacterium* spp. (Additional file 3: Table S1). Standard curve preparation and reaction conditions were carried out as described by Dostal et al. [59] using a reaction volume of 25 μl and an ABI PRISM 7500-PCR sequence detection system (Applied Biosystems, Zug, Switzerland). All assays were carried out using the 2 × SYBR Green PCR Master Mix (Applied Biosystems).

*B. thermophilum* enumeration was performed using primers bthermRTF and bthermRTR and the Taqman probe bthermTqm (Additional file 3: Table S1) [60]. The RT-QP2X-03WOULR Mastermix (Eurogentec s.a., Seraing, Belgium) was used and standard curve preparation and reaction conditions were carried out as described previously [59,60].

### Pyrosequencing

Effluent samples of CR and TRs from periods 2–4 were analyzed using 454 pyrosequencing on the V5-V6 region of the entire 16S rRNA gene pool. Reactor effluents from two consecutive days during the N-15 challenge (day 3 and 4) were pooled in a ratio 1:1, prior to DNA extraction using the FastDNA SPIN Kit for soil (MP Biomedicals). Genomic DNA extracts were sequenced by DNAVision SA (Charleroi, Belgium) on a 454 Life Sciences Genome Sequencer GS FLX instrument (Roche AG, Basel, Switzerland), and subsequent taxonomic assignment of the 16S rRNA gene reads was done as described previously [61]. Quality-filtered sequencing reads were assigned using the Ribosomal Database Project (RDP) Bayesian classifier (v 2.1) [62] and applying a confidence threshold of 80%. The entire 454 pyrosequencing dataset has been deposited to the National Center for Biotechnology (NCBI) Sequence Read Archive under accession number SRP044728.

### Metabolite analysis

Reactor effluents were analyzed for SCFAs (acetate, propionate and butyrate), BCFAs (valerate, *iso-*valerate and *iso-*butyrate), formate and lactate by HPLC (Thermo Fisher Scientific Inc. Accela, Wohlen, Switzerland) [33]. Effluent samples were centrifuged (14 000 × g, 10 min, 4°C); the resulting supernatant was diluted 1:10 with ultrapure water and directly filtered through a 0.45 μm nylon filter (Infochroma AG, Zug, Switzerland). The analysis was carried out using an Aminex HPX-87H column (Bio-Rad Laboratories AG, Reinach, Switzerland) and 10 mM H_2_SO_4_ as eluent. Mean metabolite concentrations (mM) were estimated from duplicate analyses. Total SCFA contents correspond to the sum of acetate, propionate and butyrate.

### Statistical analysis

All statistical analyses were performed using JMP 10.0 (SAS Institute Inc., Cary, NC). Prior to statistical analysis qPCR data were log_10_ transformed. HPLC and qPCR data are expressed as means ± SD from three consecutive days (days 2–4) during N-15 challenge periods. Metabolite and qPCR data from each treatment reactor were compared pairwise to the control reactor within the same period using the non-parametric Kruskal-Wallis Test. P-values <0.05 were considered significant.

## Additional files

Additional file 1: Figure S1. Microbial composition in reactors during treatment periods 2–4 measured by 454 pyrosequencing on phylum level. Microbial composition in reactor effluents was analyzed by 454 pyrosequencing of the entire 16S rRNA gene pool in the V5-V6 region. Reactor effluents were pooled in a ratio 1:1 from two consecutive days of the N-15 challenge period (days 3 and 4) for genomic DNA extraction and subsequent sequencing on a 454 Life Sciences Genome Sequencer GS FLX instrument. Quality-filtered sequencing reads were assigned using the Ribosomal Database Project (RDP) Bayesian classifier (v2.1) and applying a confidence threshold of 80%. CR: control reactor; values <1% are summarized in the group others. Additional file 2: Figure S2. Microbial composition in reactors during treatment periods 2–4 measured by 454 pyrosequencing on genus level. Microbial composition in reactor effluents was analyzed by 454 pyrosequencing of the entire 16S rRNA gene pool in the V5-V6 region. Reactor effluents were pooled in a ratio 1:1 from two consecutive days of the N-15 challenge period (days 3 and 4) for genomic DNA extraction and subsequent sequencing on a 454 Life Sciences Genome Sequencer GS FLX instrument. Quality-filtered sequencing reads were assigned using the Ribosomal Database Project (RDP) Bayesian classifier (v2.1) and applying a confidence threshold of 80%. CR: control reactor; values <1% are summarized in the group others. Additional file 3: Table S1. Primers and probes used for detection of bacterial target groups with qPCR.

## Abbreviations

RBL67: *Bifidobacterium thermophilum* RBL67
N-15: *Salmonella enterica* subsp. *enterica* serovar Typhimurium N-15
FOS: Fructooligosaccharide
GOS: Galactooligosaccharide
MOS: Mannanoligosaccharide
IR: Inoculum reactor
CR: Control reactor
TR: Test reactor
CN: Gene copy numbers
R-FOS: RBL67 + FOS
R-GOS: RBL67 + GOS
R-MOS: RBL67 + MOS
SCFA: Short chain fatty acids
BCFA: Branched chain fatty acids
qPCR: Quantitative PCR
BLIS: Bacteriocin-like inhibitory substance
