# Supplementary figures and images for: Synergistic effects of Bifidobacterium thermophilum RBL67 and selected prebiotics on inhibition of Salmonella colonization in the swine proximal colon PolyFermS model
Source: Gut Pathog. 2014 Oct 24;6:44. doi: 10.1186/s13099-014-0044-y (PMC4215022; doi:10.1186/s13099-014-0044-y)

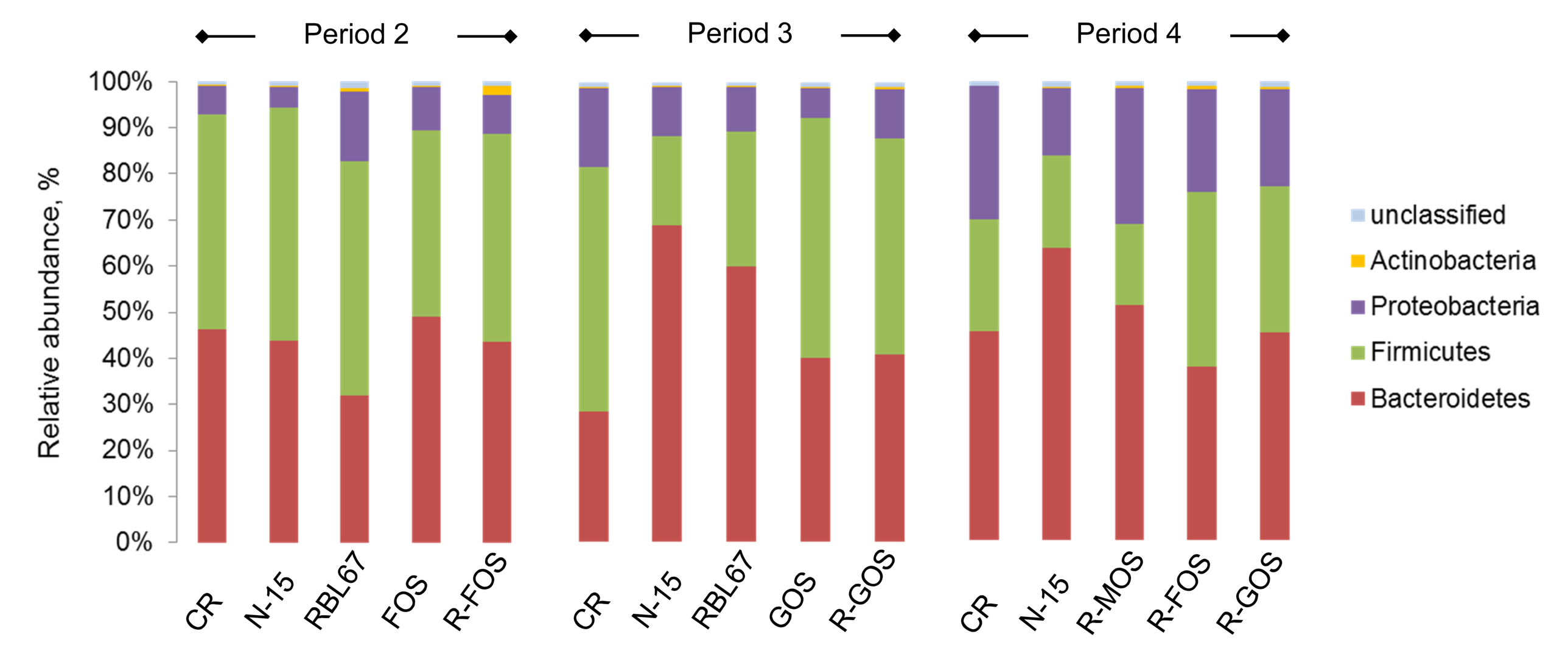

Supplement: Additional file 1: Figure S1. — Microbial composition in reactors during treatment periods 2–4 measured by 454 pyrosequencing on phylum level. Microbial composition in reactor effluents was analyzed by 454 pyrosequencing of the entire 16S rRNA gene pool in the V5-V6 region. Reactor effluents were pooled in a ratio 1:1 from two consecutive days of the N-15 challenge period (days 3 and 4) for genomic DNA extraction and subsequent sequencing on a 454 Life Sciences Genome Sequencer GS FLX instrument. Quality-filtered sequencing reads were assigned using the Ribosomal Database Project (RDP) Bayesian classifier (v2.1) and applying a confidence threshold of 80%. CR: control reactor; values <1% are summarized in the group others. [file 13099_2014_44_MOESM1_ESM.tiff]

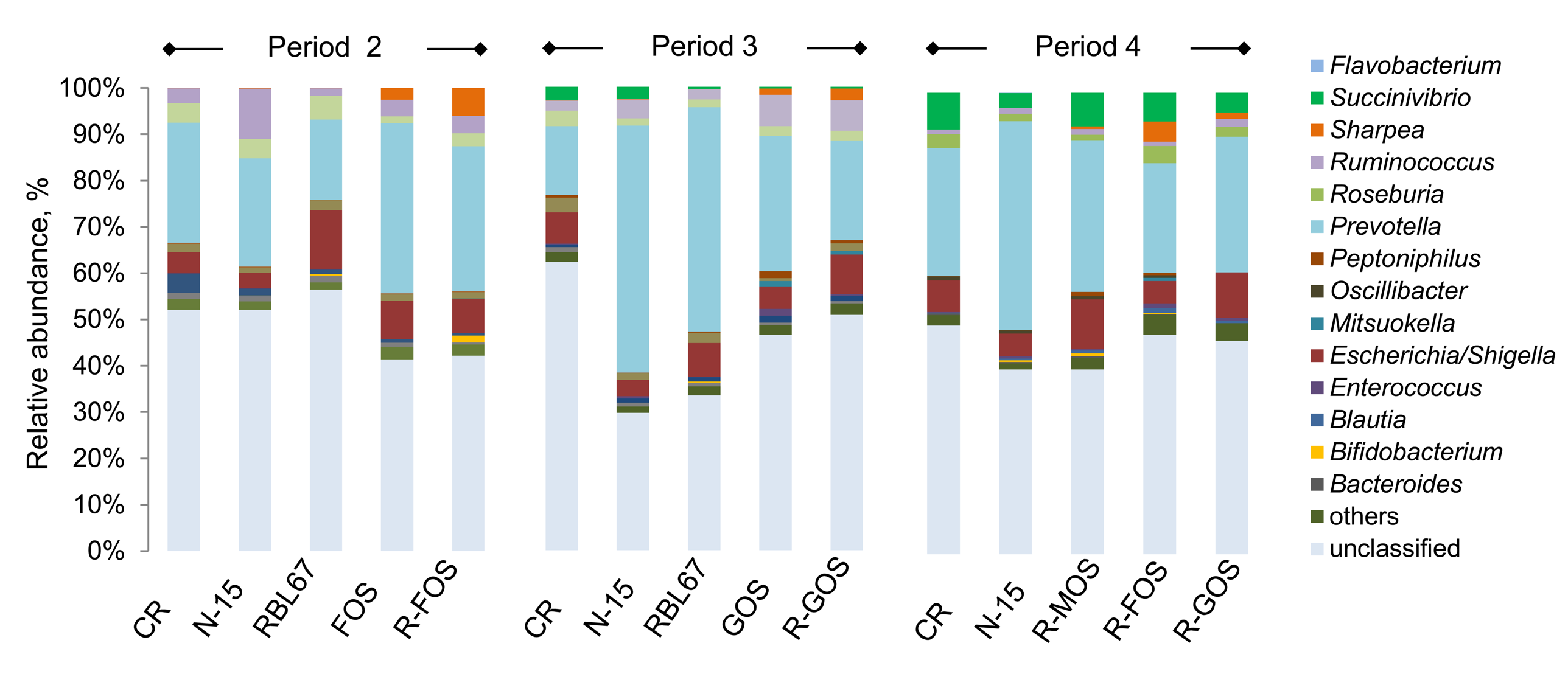

Supplement: Additional file 2: Figure S2. — Microbial composition in reactors during treatment periods 2–4 measured by 454 pyrosequencing on genus level. Microbial composition in reactor effluents was analyzed by 454 pyrosequencing of the entire 16S rRNA gene pool in the V5-V6 region. Reactor effluents were pooled in a ratio 1:1 from two consecutive days of the N-15 challenge period (days 3 and 4) for genomic DNA extraction and subsequent sequencing on a 454 Life Sciences Genome Sequencer GS FLX instrument. Quality-filtered sequencing reads were assigned using the Ribosomal Database Project (RDP) Bayesian classifier (v2.1) and applying a confidence threshold of 80%. CR: control reactor; values <1% are summarized in the group others. [file 13099_2014_44_MOESM2_ESM.tiff]
