# Supplementary material for: Synergistic effects of Bifidobacterium thermophilum RBL67 and selected prebiotics on inhibition of Salmonella colonization in the swine proximal colon PolyFermS model
Source: Gut Pathog. 2014 Oct 24;6:44. doi: 10.1186/s13099-014-0044-y (PMC4215022; doi:10.1186/s13099-014-0044-y)
Supplement: Additional file 3: Table S1. — Primers and probes used for detection of bacterial target groups with qPCR. [file 13099_2014_44_MOESM3_ESM.pdf]

Table S1. Primers and probes used for detection of bacterial target groups with qPCR

**Target**	**Primer/Probe**	**Sequence 5'-3'**	**Reference**
total 16S rRNA genes	Eub338F<br>Eub518R	ACT CCT ACG GGA GGC AGC AG<br>ATT ACC GCG GCT GCT GG	[1]
Bifidobacterium spp.	xfp-fw<br>xfp-rv	ATC TTC GGA CCB GAY GAG AC<br>CGA TVA CGT GVA CGA AGG AC	[2]
Lactococcus/ Pediococcus/ Leuconostoc spp.	F_Lacto 05<br>R_Lacto 04	AGC AGT AGG GAA TCT TCC A<br>CGC CAC TGG TGT TCY TCC ATA TA	[3]
Bacteroides-*Prevotella*	Bac303F<br>Bfr-Femrev	GAA GGT CCC CCACAT TG<br>CGC KAC TTG GCT GGT TCA G	[4]
Enterobacteriaceae	Eco1457F<br>Eco1652R	CAT TGA CGT TAC CCG CAG AAG AAG C<br>CTC TAC GAG ACT CAA GCT TGC	[5]
Clostridium Cluster IV	Clep866mF<br>Clep1240mR	TTA ACA CAA TAA GTW ATC CAC CTG G<br>ACC TTC CTC CGT TTT GTC AAC	[4]
Bifidobacterium thermophilum	btherm RTF<br>btherm RTR<br>bthermTqm	TTG CTT GCG GGT GAG AGT<br>CGC CAA CAA GCT GAT AGG AC<br>FAM-ATG TGC CGG GCT CCT GCA T-TAMRA	[6]

References for Additional file 1: Table S1

1. Guo X, Xia X, Tang R, Zhou J, Zhao H, Wang K: **Development of a real-time PCR method for *Firmicutes* and *Bacteroidetes* in faeces and its application to quantify intestinal population of obese and lean pigs.** *Lett Appl Microbiol* 2008, **47:**367-373.

2. Cleusix V, Lacroix C, Dasen G, Leo M, Le Blay G: **Comparative study of a new quantitative real-time PCR targeting the xylulose-5-phosphate/fructose-6-phosphate phosphoketolase bifidobacterial gene (*xfp*) in faecal samples with two fluorescence *in situ* hybridization methods.** *J Appl Microbiol* 2010, **108:**181-193.

3. Furet JP, Firmesse O, Gourmelon M, Bridonneau C, Tap J, Mondot S, Dore J, Corthier G: **Comparative assessment of human and farm animal faecal microbiota using real-time quantitative PCR.** *FEMS Microbiol Ecol* 2009, **68:**351-362.

4. Ramirez-Farias C, Slezak K, Fuller Z, Duncan A, Holtrop G, Louis P: **Effect of inulin on the human gut microbiota: stimulation of *Bifidobacterium adolescentis* and *Faecalibacterium prausnitzii*.** *Br J Nutr* 2009, **101:**541-550.

5. Bartosch S, Fite A, Macfarlane GT, McMurdo ME: **Characterization of bacterial communities in feces from healthy elderly volunteers and hospitalized elderly patients by using real-time PCR and effects of antibiotic treatment on the fecal microbiota.** *Appl Environ Microbiol* 2004, **70:**3575-3581.

6. Mathys S, Lacroix C, Mini R, Meile L: **PCR and real-time PCR primers developed for detection and identification of *Bifidobacterium thermophilum* in faeces.** *BMC Microbiol* 2008, **8:**179.
